# Three Day Environmental Exposure May Trigger Oxidative Stress Development and Provoke Adaptive Response Resulting in Altered Antioxidant Activity

**Published:** 2019-07

**Authors:** Zlatko ZIMET, Marjan BILBAN, Joško OSREDKAR, Borut POLJŠAK, Teja FABJAN, Kristina SUHADOLC

**Affiliations:** 1. National Institute of Public Health, Ljubljana, Slovenia; 2. Department of Public Health, Faculty of Medicine, University of Ljubljana, Ljubljana, Slovenia; 3. Institute of Clinical Chemistry and Biochemistry, University Medical Center Ljubljana, Ljubljana, Slovenia; 4. Laboratory of Oxidative Stress Research, Faculty of Health Sciences, University of Ljubljana, Ljubljana, Slovenia

**Keywords:** Polluted working environment, Oxidative stress, 8-isoprostane, Enzymatic antioxidant activity

## Abstract

**Background:**

We aimed to investigate the polluted working environment triggers oxidative stress and alter enzymatic antioxidant activity by a short-term interval.

**Methods:**

The experimental study, performed in 2014, involved 94 workers from the Velenje Coalmine in Slovenia, arranged into three groups according to a number of consecutive working days in a mineshaft, supported by a control group. Levels of the antioxidant enzymes (GPx, CAT, SOD) together with TAC (the combined effect of all antioxidants) and 8-isoprostane (a biological marker of oxidative stress/damage) were measured in human plasma.

**Results:**

Workers occupationally exposed for three consecutive working days had significantly increased 8-isoprostane biomarker, a parameter of oxidative stress (*P*<0.001). The antioxidant levels of TAC (*P*<0.001), CAT (*P*<0.001) and SOD (*P*<0.001) were all significantly decreased compared to a control group.

**Conclusion:**

Workers in polluted working environment had significantly increased oxidative stress and altered antioxidant activity already on a third consecutive working day.

## Introduction

Several studies suggest that polluted working environment involves oxidative damage (1–5). Oxidative stress can increase the risk of diseases like chronic obstructive pulmonary disease, arterial hypertension, diabetes, cancer and in general premature aging (6). Slovenian mining industry has in 2015, in comparison to other industrial fields, lost most working days because of respiratory disease, gastrointestinal disease, eye disease and skin disorders (7).

Oxidative stress is a consequence of an increased generation of free radicals, where environmental pollutants stimulate a variety of mechanisms of toxicity on molecular level as well as modulation of antioxidant enzymes (8, 9). Moreover, the lowered antioxidant level may compromise one’s ability to scavenge free radicals and oxygen reactive species (ROS), which might predispose them to disease formation (10, 11). The human cell balances the potential danger of ROS by the antioxidant reserve used to oppose to the ROS diffusion and to limit the propagation process. The antioxidant reserve is primarily composed of a combination of damping ROS enzymes and the non-enzymatic antioxidants (12). The first enzymatic system involved in ROS production is the superoxide dismutase (SOD). As common characteristics, all SODs accelerate thousands of times the transformation in O2^•−^ and H_2_O_2_. Catalase (CAT) decomposes hydrogen peroxide to water. Glutathione peroxidase (GPx) reduces lipid hydroperoxides to alcohols and hydrogen peroxide to water. This process involves also an inactivation and elimination of harmful substances to the body and it is one of the main mechanisms of protection of exposed organs (lung) or ones interested in the detoxification (liver) (13).

The 8-isoprostane is a prostaglandin (PG)-F2-like compound and represents the major F2 isoprostane. It is produced in vivo by the free radical-catalyzed peroxidation of arachidonic acid and its metabolites can be routinely measured in various body fluids, e.g. blood plasma, urine and exhaled breath condensate (14). The 8-isoprostane in plasma was used many times before to indicate oxidative status development in polluted working environment (15–17). Moreover, oxidative stress was studied in connection to the total antioxidant capacity (TAC) as well as SOD, CAT and GPx enzymatic activity (4, 18–20). Past studies had more or less specific study designs and conducted results were in some contradiction. For example, vitamin E and C absorption resulted in increased TAC, GPx and SOD, CAT was reduced (18). Workers also absorbed vitamins E and C, which provoked reduced TAC, SOD and CAT, GPx remained unchanged (4). Nevertheless, both proved antioxidant activity in environmentally exposed workers only, control groups remained without visible change.

We aimed to investigate whether short period of work in environment with elevated concentration of dust and gases might result in the increased oxidative stress and provoke antioxidant response.

## Materials and Methods

### Study design and subjects

Experimental study design was used (nonrandomized control group pretest-posttest design (21)). Data were collected, from 850 employees of the Velenje Coalmine, Slovenia, in 2014. All were invited to participate in the study and 169 male coal miners agreed to participate. The health condition of each worker was confirmed by his general practitioner who provided written confirmation that worker’s health status is adequate: a) non-smoker, b) without chronic diseases, and c) without any drug therapy. Finally, 94 coal miners passed the above inclusion criteria.

This study was approved by the Slovenian National Ethical Committee. The involved coalmine workers signed written informed consent after being completely aware of the settings of the study.

**Fig. 1:**
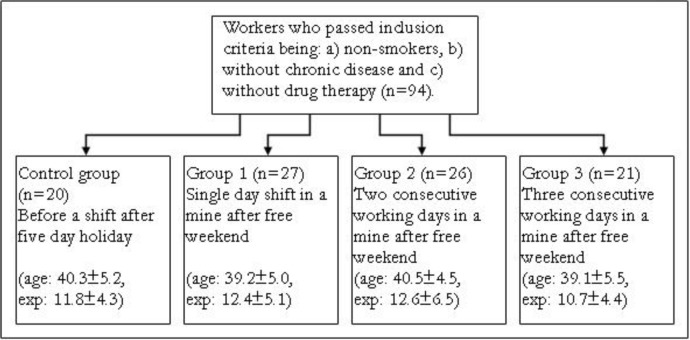
Study design and sampling (age: age in years, exp: number of working years)

The impact of polluted environment on oxidative stress of exposed workers was measured according to the number of working days in the mine (Fig. 1). The first group included workers for a single day following a free weekend (n=27). The second group included workers for two consecutive days (n=26). The third group included workers for three consecutive days (n=21). Measurements were carried out on Monday, Tuesday and Wednesday after the shift in the coalmine. Control group included workers measured before the shift after five day holiday (n=20). The control and three experimental groups were arranged according to the Velenje coal mining working schedules.

The effect of oxidative stress was measured by 8-isoprostane and enzymatic antioxidant activity by GPx, CAT, SOD and TAC (the combined effect of all antioxidants).

### 8-isoprostane in plasma

Blood EDTA samples were obtained from the antecubital vein. Plasma was, supplemented with 0.005% butylated hydroxytoluene and frozen at −80 °C till the analysis. The samples were then analyzed by the competitive enzyme immunoassay technique for the quantification of 8-isoprostane (8-Isoprostane EIA KIT, Cayman Chemical Company, USA) in accordance with the manufacturer’s instructions.

### TAC

We determined total antioxidant status in serum with Randox TAC assay (Randox Laboratories Limited, Crumlin, UK). The assay principle is as follows: ABTS (2, 2′-azino-di-[3-ethylbenzthiazoline sulphonate]) is incubated with peroxidise (metmyoglobin) and H_2_O_2_ to produce radical cation ABTS*^+^. This product has a relatively stable blue-green color, measured at 600 nm. Antioxidants in the added sample cause suppression of this color production to a degree, which is proportional to their concentration.

### CAT activity assays

Catalase activity was determined by Bioxytech Catalase-520 (OXIS). The first step is hemolysis of the sample and then application on the Olympus AU 400. The procedure is done as specified in the protocol for Olympus AU 400.

### GPx activity assays

For the quantitative determination of Glutathione Peroxidase (GPx) in whole blood, we used RANSEL Glutathione Peroxidase (Randox). GPx catalyses the oxidation of Glutathione (GSH) by Cumene hydroperoxide. In the presence of Glutathione Reductase (GR) and NADPH, the oxidized Glutathione (GSSG) is immediately converted to the reduced form with concomitant oxidation of NADPH to NADP^+^. The decrease in absorbance is measured at 340 nm.

### SOD activity assays

The method RANSOD Superoxide dismutase (Randox) employs xanthine and xanthine oxidase (XOD) to generate superoxide radicals which react with 2-(4-iodophenyl)-3-(4-nitrophenol)-5-phenyltetrazolium chloride (I.N.T.) to form a red formazan dye. The superoxide dismutase activity is then measured by the degree of inhibition of this reaction. One unit of SOD is that which causes a 50% inhibition of the rate of reduction INT under the conditions of the assay. The absorbance is measured at 505 nm.

### Statistical analysis

Results were expressed as mean ± standard deviation. Differences between groups were compared using independent samples t-test. The one-way analysis of variance was used to analyze groups by age and working experience. Shapiro-Wilk test has confirmed normal distribution of data. Statistical analysis was made with IBM SPSS 22.0 software (IBM Corp., Armonk, NY). Statistical significance was assumed at *P*<0.05. All analyzed demographic characteristics and biomarkers were without missing data.

## Results

Mineworkers were 39.2±4.9 yr of age and had 11.6±4.8 yr of working experiences in the coalmine. All workers were resident in rural areas in the surrounding of the Velenje coalmine. Selected groups of workers were not significantly different in their age (C: 40.3±5.2, G1: 39.2±5.0, G2: 40.5±4.5; G3: 39.1±5.5, *P*=0.642) and number of working years in the coalmine (C: 11.8±4.3, G1: 12.4±5.1, G2: 12.6±6.5, 10.7±4.4, *P*=0.496). All workers graduated from a four years mining school, followed by a 14-d on-site training. After 6 months of mentorship, miners start to work on their own. During mining career, they are obliged to attend annual education about mining safety and mining technology in order to keep their license.

**Fig. 2:**
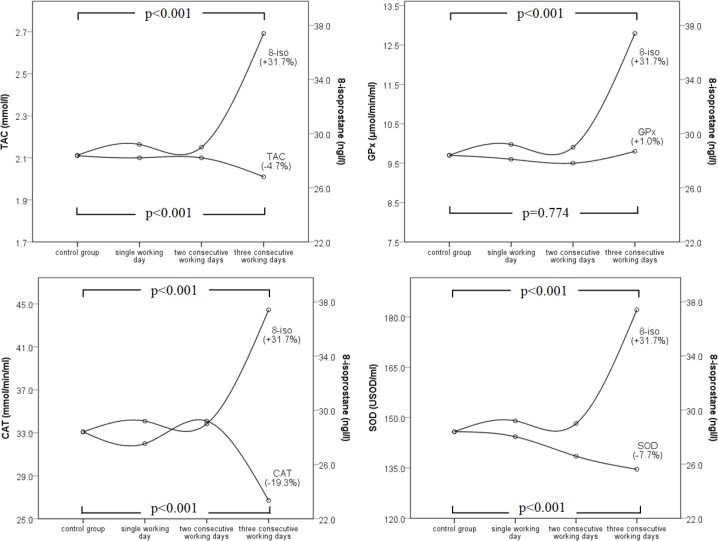
TAC, GPx, CAT, SOD status and oxidative stress development in workers arranged by consecutive working days

Workers in group 3 occupationally exposed for three consecutive working days had significantly increased oxidative stress (8-isoprostane biomarker, *P*<0.001). Among antioxidant activity biomarkers TAC (*P*<0.001), CAT (*P*<0.001) and SOD (*P*<0.001) were all significantly decreased compared to control group (Table 1). Solely GPx remained unchanged (*P*=0.744). Figure 2 shows that oxidative stress increase resulted in antioxidant biomarkers decrease, vertical spread between the curves was evident on a third consecutive working day.

**Table 1:** Biomarkers of oxidative stress and antioxidant activity

***Biomarker***	***Control group (n=20)***	***Group 3 (n=21)***	P^*^
8-isoprostane (ng/l)	28.4±5.9	37.4±4.9	<0.001
TAC (mmol/l)	2.11±0.06	2.01±0.03	<0.001
GPx (μmol/min/ml)	9.7±2.7	9.8±1.3	0.744
CAT (mmol/min/ml)	33.1±6.9	26.7±5.9	<0.001
SOD (USOD/ml)	145.8±5.4	134.6±3.6	<0.001

* Independent samples t-test

Typical coalmine environmental pollutants are represented by coal dust mixture with CH_4_ (22) and CO_2_ (23) gases. Mean concentrations of CH_4_ (%) and CO_2_ (%) in the coal mine shafts during the three days of research were 0.25±0.07 and 0.52±0.17, respectively. Both values show increased gas exposure for workers in comparison to the outdoor air in the courtyard (0.11±0.03 and 0.08±0.03, respectively; *P*<0.001).

## Discussion

Polluted working environment represents high health risk, on the other hand, physiological processes that take part in human body are less certain, especially in short term intervals. Our study concentrated on short-term antioxidant activity and did not involve an intervention to uphold antioxidant levels; workers were on normal food, for example without vitamin supplements and without any instructions for altered daily activities. Workers on a third consecutive working day had significantly elevated oxidative stress, which resulted in significantly decreased levels of antioxidant biomarkers.

Scientific research results are difficult to compare as each study has a more or less specific study design, furthermore, there is insufficient provision of interim measurements during the entire study duration and it is therefore impossible to conclude which processes took part in this time. In case of larger study time intervals authors should provide more interim results and not only before start (first) and after the result. We tried to provide some insight on how human body in polluted working environment reacts on short term. On the third consecutive working day, 8-isoprostane increased, TAC, CAT, and SOD decreased, interpreted because of oxidative stress. Solely GPx remained unchanged. A similar picture was presented in a factory with dust mixture of lead oxides (4) and in a coalmine, yet in the latter research, GPx reacted too (18). Comparison of the results is also handicapped by the range of the reactions of enzymatic antioxidants and TAC, from a single digit percentage towards 100% or more. Yet this might be reasonable because different working environments provide different circumstances for physiological processes in a human body.

Scholar explanation of TAC proves confusing, as for example, workers in factory with dust mixture of lead oxides had higher TAC (4), on the other hand, cement plant workers had lower TAC (24). In this context, it is difficult to define the state of the initial TAC value or in other words, higher TAC being favourable or unfavourable for once health. In that manner, lower TAC is considered as increased production of free radicals (24–26) and higher TAC as a consequence of long term exposure and a result of adaptive stress responses (4, 18). Yet there seems to exist common agreement that antioxidant activity (in positive or negative direction) is influenced by environmental pollutants (1, 3, 27, 28). This phenomenon was proven by inclusion of control group, where during the experiment no antioxidant activity was registered (4, 18). Our results showed significant antioxidant activity already on a third consecutive working day, other studies that involved experiments were very durable and lack of interim results, it is therefore unclear at what stage the antioxidant activity was neutralized. Therefore, one can argue by how long and by which frequency interventions are needed so that workers are not exposed to production of free radicals.

Redox regulation represents a very complex mechanism where it is impossible to understand fully the entire event cascade algorithm. It is clear, however, the oxidative allostasis is balanced between ROS production/reactivity and ROS protective mechanisms and the redox-responsive transcription system represents an essential part of allostasis (12). What remains unclear is at what stage/level and time particular oxidative stress-inducing compound trigger adaptive cellular responses resulting in increased antioxidant defence and repair.

This study objective was to explore how short term environmental exposure affects occupational workers. We measured 8-isoprostane as an oxidative stress biomarker in human plasma and enzymatic antioxidant activity by GPx, CAT, SOD, and TAC in coalmine workers. Our results show that workers occupationally exposed for three consecutive working days had significantly increased oxidative stress (8-isoprostane biomarker, *P*<0.001). Among antioxidant activity biomarkers TAC (*P*<0.001), CAT (*P*<0.001) and SOD (*P*<0.001) were all significantly decreased compared to control group. This may suggest that environmental exposure is also relevant on short (few days) terms, not just on longer (few weeks or months) terms as conducted in previous studies.

### Limitations

Our intention was to arrange three groups of at least 30 workers. Since we were obliged not to interfere into working process, we could only follow 47 workers after first working day, as 16 workers unexpectedly received other assignments and did not spend their entire second consecutive working day in the coalmine under increased air pollution. Anticipated control group was also reduced to 20 workers, as 10 among them had to interrupt their holidays. Since the study participants were without registered health problems, non-smokers and the groups were matched according to age and working years, the differences in antioxidant activity clearly represent the influence of longer occupational exposure to pollutants by consecutive working days.

To reduce influence of body fluids loss we measured workers who controlled heavy mining equipment for excavation of coal only. They did not installed rails for transportation or performed another sort of strenuous physical work. In addition, study participants were addressed to maintain usual food intake, avoid strenuous daily activities and social events to omit passive smoking and alcohol consumption during the three-day research period. Regarding their living area participants stated that family members or visitors smoke outside the premises or on the balcony.

Our control and experimental groups were controlled in basic demographic and health characteristics (age, number of working years, education, residence, nonsmoking status, no chronic disease, and drug prescription). Moreover, advice was given to avoid passive smoking and alcohol consumption and to perform only moderate daily activities. Certain inability to control for above-listed confounding variables (e.g. disregard of advice to avoid passive smoking, alcohol consumption and strenuous daily activities), may have influenced our results of oxidative stress development.

Sources of bias were reduced as researches could not influence sampling, all employed miners were asked to join the research by the mining company, n=169 agreed to participate. Part of the participants was rejected as they did not pass the inclusion criteria (n=40), some reported ill (n=9), some unexpectedly received other assignments (n=16), some were recalled from the holiday (n=10) and the rest (n=94) eventually took part. Researchers also could not influence on control and experimental groups formation because all the development was scheduled by the mining company. Regarding the analysis of biological samples laboratory staff was not aware of control and experimental groups’ formation. All methods used, were routine laboratory methods and were validated and performed upon manufacturer instructions. For all methods, specially designed quality control material was used. The inter- and intra- assay coefficients of variation were within the limits proposed by the manufacturer.

Since this study was composed of miners, results may not generalize to other occupational groups. In addition, concentrations of various metallic compounds in the gas/dust mixture of the breathing air may also compromise applicability and generalisability of results to other international mining areas.

## Conclusion

Our sample of workers in polluted working environment had significantly increased oxidative stress and altered antioxidant activity already on a third consecutive working day. Different scientific approaches reveal more and more information on what to consider about oxidative stress to alleviate its impact on people working in polluted environments.

## Ethical considerations

Ethical issues (including plagiarism, informed consent, misconduct, data fabrication and/or falsification, double publication and/or submission, redundancy, etc.) have been completely observed by the authors.
